# Fat mass to fat-free mass ratio and the risk of non-alcoholic fatty liver disease and fibrosis in non-obese and obese individuals

**DOI:** 10.1186/s12986-021-00551-6

**Published:** 2021-02-19

**Authors:** Huajie Dai, Jiali Xiang, Yanan Hou, Liping Xuan, Tiange Wang, Mian Li, Zhiyun Zhao, Yu Xu, Jieli Lu, Yuhong Chen, Weiqing Wang, Guang Ning, Yufang Bi, Min Xu

**Affiliations:** 1 Department of Endocrine and Metabolic Diseases, Shanghai Institute of Endocrine and Metabolic Diseases, Ruijin Hospital, Shanghai Jiao Tong University School of Medicine, 197 Ruijin 2nd Road, Shanghai, 200025 China; 2 Shanghai National Clinical Research Center for Metabolic Diseases, Key Laboratory for Endocrine and Metabolic Diseases of the National Health Commission of the PR China, Shanghai National Center for Translational Medicine, Ruijin Hospital, Shanghai Jiao Tong University School of Medicine, Shanghai, China

**Keywords:** Fat-to-fat free mass ratio, Non-alcoholic fatty liver disease, Liver fibrosis, Mediation analysis, Prospective investigation

## Abstract

**Context:**

Body composition may explain partially why non-obese individuals still at the risk of developing non-alcoholic fatty liver disease (NAFLD). The ratio of fat mass to fat-free mass (FM/FFM) has been proposed to assess the combined effect of different body compositions.

**Objective:**

We aimed to investigate the associations of FM/FFM ratio with the risk of developing NAFLD and fibrosis and to identify the potential mediators according to obesity status.

**Methods:**

This cohort study comprised 3419 adults age ≥ 40 years and free of NAFLD at baseline. Body composition was measured by bioelectrical impedance analysis. NAFLD was ascertained by ultrasonography and fibrosis was assessed by non-invasive score systems.

**Results:**

For each 1 standard deviation increment in FM/FFM ratio, the odds ratio for the risk of NAFLD was 1.55 (95% confidence interval [CI] 1.23–1.95) in non-obese men, 1.33 (95% CI 1.08–1.65) in obese men, 1.42 (95% CI 1.44–1.67) in non-obese women, and 1.29 (95% CI 1.12–1.50) in obese women. Similar associations were also found between FM/FFM ratio and NAFLD with fibrosis. Mediation analysis showed that insulin resistance, triglycerides, high-density lipoprotein cholesterol, white blood cells, and total cholesterol mediated the association of FM/FFM ratio with NAFLD risk in specific sex and obesity subgroups.

**Conclusions:**

The FM/FFM ratio significantly associated with the NAFLD and fibrosis risk in both non-obese and obese individuals. Different factors may mediate the association between body composition and NAFLD risk according to different obesity status.

## Introduction

Non-alcoholic fatty liver disease (NAFLD) encompasses a spectrum of liver-lipid associated liver conditions ranging from excess fat deposits in the liver (steatosis) to the more aggressive nonalcoholic steatohepatitis (NASH), which is characterized by hepatic inflammation (steatohepatitis) that prompts fibrosis of liver tissue [1]. Compelling data demonstrate that NAFLD is an established risk factor for cardiovascular diseases, type 2 diabetes (T2D), and some kinds of cancers as well [2]. It has become the most common chronic liver disease in the world, with global prevalence was currently estimated to be 25.2% [3]. Hence, more efforts are urgently needed on the acquirement of knowledge about the prevention and management of NAFLD and ultimately help mitigate its global impact.

Although NAFLD has been strongly associated with obesity, a proportion of cases has a normal body mass index (BMI) [4, 5]. This phenomenon is termed as “non-obese NAFLD” or “lean NAFLD”, and its worldwide prevalence is also growing substantially [5, 6]. Some studies suggested that these patients had higher mortality and accelerated disease progression despite a less severe metabolic phenotype [6, 7]. However, there was little information on pathogenesis, treatment, even screening of NAFLD in non-obese patients [8].

BMI is the most commonly used indicator to assess obesity. However, BMI has one major shortcoming, which is that BMI does not account for body composition [9]. Individuals with the same BMI may vary in body composition largely. Recent studies showed that different compositions including fat mass and fat-free mass might play different roles in health outcomes. Large prospective cohort studies showed that increased fat mass could significantly increase the risk of T2D, cardiovascular disease, even mortality [10–12]. In contrast, an increment in fat-free mass reduced the risks of these conditions [12–14]. Therefore, BMI might not be a sufficient indicator assessing obesity-related health risks. Worse body composition in non-obese individuals classified by BMI might have a hand in the occurrence and development of NAFLD. The body composition in lean patients with NAFLD was deemed as a possible essential contributor to the development of the disease phenotype [15].

The ratio of fat mass to fat-free mass (FM/FFM) has been proposed as a potential novel indicator to assess the combined effect of different body compositions. Prado CMM et al. proposed that FM/FFM ratio represented metabolic load/capacity model, using fat mass as the agent of metabolic load, defined as the magnitude of an insult on a system, and fat-free mass as the agent of metabolic capacity, defined as the ability of the system to counteract the insult [16]. The higher FM/FFM ratio has also been proposed as an alternative definition of sarcopenic obesity, which was characterized as a confluence of sarcopenia and obesity [17].

There were several studies that investigated the associations of FM/FFM with health outcomes and found that the ratio was associated with insulin resistance, liver fat accumulation, glucose metabolic disorders, and metabolic syndrome [18–20]. However, the association of the FM/FFM ratio with the risk of incident NAFLD and fibrosis remained unclear, especially whether it could predict the NAFLD risk in non-obese individuals has not been estimated.

The aim of this study was mainly to evaluate in non-obese and obese individuals separately: (1) the association between the FM/FFM ratio and the risk of incident NAFLD and fibrosis; (2) whether it is the same metabolic or inflammatory factors mediated the association between FM/FFM ratio and NAFLD.

## Methods

### Study population

Participants were recruited from a community-based cohort study as reported previously [21]. In brief, between March to August 2010, 10,375 residents aged 40 years or older living in Jiading district, Shanghai, China, were invited and received a comprehensive health survey, which included a structured questionnaire and relevant clinical measurements. Of these participants, we excluded subjects who (a) did not complete abdominal ultrasound evaluation (n = 45); (b) diagnosed as having NAFLD by abdominal ultrasound (n = 2687); (c) with self-reported history of viral and autoimmune hepatitis, cirrhosis, or hepatic malignancy (n = 378); (d) consumed alcohol of ≥ 140 g/week for men and ≥ 70 g/week for women (n = 980); (e) with missing data on body composition (n = 1244). Finally, 5635 participants were eligible for this prospective investigation. From August 2014 to May 2015, we invited the remaining 5635 participants to attend a follow-up visit. In the current analysis, we further excluded participants who did not attend a follow-up visit (n = 2039) or had missing data on abdominal ultrasonography (n = 177). Finally, 3419 participants were included in this study.

The study protocol was approved by the Institutional Review Board of Ruijin Hospital affiliated to the Shanghai Jiaotong University School of Medicine and procedures were in accordance with the ethical standards of the responsible committee on human experimentation and with the Helsinki declaration of 1975, as revised in 1983. Written informed consent was obtained from each participant before data collection.

### Data collection

Trained personnel performed data collection according to a standard protocol at baseline and follow-up visits. A standard questionnaire was used to collect demographic characteristics, as well as medical history and lifestyle factors (including cigarette smoking and alcohol drinking). Besides, we acquired physical activity at work and in leisure time using the short form of the International Physical Activity Questionnaire (IPAQ).

Body weight, height, waist circumference, and blood pressure were measured according to a standard protocol and BMI was calculated as the weight in kilograms divided by height in meters squared.

All participants underwent an oral glucose tolerance test after an overnight fast of at least 10 h, blood samples were collected at 0 and 2 h during the test. Plasma glucose level was measured using the glucose oxidase method on an auto-analyzer (Modular P800; Roche, Basel, Switzerland). Serum insulin was measured by using an electro chemiluminescence assay (Modular E170, Roche, Basel, Switzerland). Insulin resistance was estimated by the homeostasis model assessment of insulin resistance (HOMA-IR) index: fasting serum insulin (μIU/mL) × fasting plasma glucose (mmol/L)/22.5. Serum triglycerides, total cholesterol, low density lipoprotein cholesterol (LDL-C), high density lipoprotein cholesterol (HDL-C), alanine aminotransferase (ALT), aspartate aminotransferase (AST), γ‐glutamyl transferase (GGT), and albumin were measured on the auto-analyzer (Modular E170, Roche, Basel, Switzerland).

Liver ultrasonography was performed by two experienced specialists using high-resolution B-mode tomographic ultrasound system (Esaote Biomedica SpA, Italy) with a 3.5-MHz probe. Body composition (fat mass and fat-free mass) was measured by bioelectrical impedance analysis (BIA) using a body composition analyzer (Tanita TBF-300, Japan).

### Definitions

Current smoking or drinking was defined as smoking cigarettes or consuming any kind of alcohol regularly in the past 6 months, respectively. Education level was categorized as less than high school and high school or further education. Active physical activity was defined as moderate intensity exercise for ≥ 150 min/week or vigorous intensity exercise for ≥ 75 min/week or moderate and vigorous physical activity for ≥ 150 min/week. Hypertension was defined as systolic blood pressure ≥ 140 mmHg, diastolic blood pressure ≥ 90 mmHg, or self-reported physician diagnosed hypertension or current use of any antihypertensive medications. Dyslipidemia was defined as total cholesterol ≥ 6.22 mmol/L, or LDL-C ≥ 4.14 mmol/L, or HDL-C < 1.04 mmol/L, or triglycerides ≥ 2.26 mmol/L, or self-reported physician diagnosed dyslipidemia or taking any lipid-lowering medications. T2D was defined as fasting plasma glucose ≥ 7.0 mmol/L, or 2-h oral glucose tolerance test plasma glucose ≥ 11.1 mmol/L, or self-reported physician diagnosis of T2D or current use of antidiabetic medications [22]. Obesity was defined as BMI ≥ 25.0 kg/m^2^ according to Asia-specific BMI criteria, which  was determined by the World Health Organization Western Pacific Region [23].

### Outcome ascertainment

NAFLD was diagnosed by ultrasonography with the presence of at least two of the following three findings: (a) diffusely increased echogenicity of the liver relative to the kidney or spleen; (b) ultrasound beam attenuation; (c) poor visualization of intrahepatic structures, after excluding those with excessive alcohol consumption and other liver diseases [24].

Non-invasive scoring systems including the NAFLD fibrosis score (NFS), and the fibrosis‐4 score (FIB-4), were used to evaluate the probability of advanced fibrosis in NAFLD patients [24]. These indices were calculated according to original reported formulas: NFS = − 1.675 + 0.037 × age (years) + 0.094 × BMI + 1.13 × impaired fasting glucose or T2D (yes = 1, no = 0) + 0.99 × ALT/AST ratio − 0.013 × platelet (× 10^9^/L) − 0.66 × albumin (g/dl); FIB-4 = age (years) × AST (U/L)/[platelet (× 10^9^/L) × ALT (U/L)^1/2^]. A higher probability of fibrosis was defined as NFS ≥ − 1.455 or FIB-4 ≥ 1.3 [25].

### Statistical analysis

Baseline characteristics of participants are summarized as means ± standard deviation or medians (interquartile range) for continuous variables and numbers (proportions) for categorical variables. Participants were divided into four groups according to sex and obesity status. For comparisons between groups within particular sex, we conducted student’s t tests for continuous variables and chi-square tests for categorical variables.

All analysis was performed by sex considering the noteworthy difference of body composition between men and women. Logistic regression models with adjustment of age, current smoking, current drinking, education level, and active physical activity were used to estimate odds ratios (ORs) and 95% confidence intervals (CIs) of the FM/FFM ratio for incident NAFLD and fibrosis among different obesity status. We further adjusted for multiple metabolic and inflammatory factors to examine whether these factors mediated the associations between FM/FFM and NAFLD and fibrosis.

The causal mediation analysis based on the counterfactual method was performed to assess the extent to which the association between FM/FFM ratio and risk of NAFLD was mediated by several metabolic and inflammatory factors [26].

Significance tests were two-tailed, with a *p* value < 0.05 considered as statistically significant. All statistical analyses were performed by using SAS software, version 9.4 (SAS Institute Inc).

## Results

### Characteristics of study participants at baseline

Of the 3419 participants in the present study, 1017 (29.7%) were men, and 2402 (70.3%) were women. 233 (22.9%) men and 488 (20.3%) women developed NAFLD during a mean follow-up of 4.4 years. Table 1 shows the general baseline characteristics of the participants according to sex and obesity status. Compared with non-obese participants, obese participants had a higher FM/FFM ratio at baseline and more NAFLD cases at follow-up in both sexes (all *p* < 0.001).

**Table 1:** Basic characteristics of study participants according to sex and obesity status

Characteristics	Men (n = 1017)			Women (n = 2402)		
	Non-obesity (n = 612)	Obesity (n = 405)	*p* value	Non-obesity (n = 1569)	Obesity (n = 833)	*p* value
Age (years)	58.7 ± 9.1	59.3 ± 9.0	0.338	56.4 ± 8.9	57.4 ± 8.1	0.006
BMI (kg/m^2^)	22.7 ± 1.7	27.1 ± 1.7	< 0.001	22.4 ± 1.8	27.1 ± 1.8	< 0.001
Waist circumference (cm)	78.9 ± 6.0	88.8 ± 5.6	< 0.001	74.8 ± 5.8	84.4 ± 5.7	< 0.001
High school education or above, n (%)	145 (23.8)	69 (17.1)	0.010	315 (20.1)	117 (14.2)	< 0.001
Lifestyle factors						
Current smokers, n (%)	308 (50.3)	184 (45.5)	0.136	8 (0.5)	4 (0.5)	0.910
Current drinkers, n (%)	33 (5.4)	21 (5.2)	0.895	8 (0.5)	5 (0.6)	0.780
Active physical activity, n (%)	141 (23.0)	74 (18.3)	0.068	294 (18.7)	149 (17.9)	0.609
Metabolic profile						
Fasting glucose (mmol/L)	5.5 ± 1.6	5.5 ± 1.4	0.896	5.2 ± 1.0	5.4 ± 1.2	< 0.001
Systolic blood pressure (mmHg)	137.1 ± 18.8	145.4 ± 18.8	< 0.001	135.5 ± 19.5	144.6 ± 20.1	< 0.001
Diastolic blood pressure (mmHg)	81.4 ± 9.3	86.0 ± 10.0	< 0.001	79.8 ± 9.9	83.8 ± 9.9	< 0.001
Total cholesterol (mmol/L)	5.0 ± 0.9	5.1 ± 0.9	0.313	5.4 ± 1.0	5.5 ± 1.0	0.001
HDL-C (mmol/L)	1.3 ± 0.3	1.2 ± 0.2	< 0.001	1.5 ± 0.3	1.4 ± 0.3	< 0.001
LDL-C (mmol/L)	3.0 ± 0.8	3.1 ± 0.8	0.063	3.2 ± 0.9	3.4 ± 0.9	< 0.001
Triglycerides (mmol/L)	1.1 (0.9–1.5)	1.4 (1.1–1.9)	0.012	1.2 (0.9–1.6)	1.4 (1.0–1.8)	< 0.001
HOMA-IR	1.1 (0.7–1.6)	1.6 (1.1–2.2)	< 0.001	1.3 (0.9–1.7)	1.7 (1.3–2.4)	< 0.001
White blood cell (10^9^/L)	6.0 ± 1.6	6.2 ± 1.5	< 0.001	5.3 ± 1.4	5.7 ± 1.5	< 0.001
Type 2 diabetes, n (%)	88 (14.4)	71 (17.5)	0.186	119 (7.6)	101 (12.1)	< 0.001
Hypertension, n (%)	321 (52.5)	278 (68.8)	< 0.001	688 (44.0)	552 (66.3)	< 0.001
Dyslipidemia, n (%)	173 (28.3)	190 (46.9)	< 0.001	475 (30.3)	312 (37.5)	< 0.001
Body composition						
Fat mass (kg)	13.1 ± 3.5	20.1 ± 4.5	< 0.001	16.2 ± 3.9	24.4 ± 4.6	< 0.001
Fat-free mass (kg)	47.7 ± 4.3	51.9 ± 5.0	< 0.001	37.1 ± 3.4	38.8 ± 3.7	< 0.001
Fat-to-fat free mass ratio	0.3 ± 0.1	0.4 ± 0.1	< 0.001	0.4 ± 0.1	0.6 ± 0.1	< 0.001
NAFLD cases, n (%)	94 (15.4)	139 (34.3)	< 0.001	216 (13.8)	272 (32.7)	< 0.001

Data were means ± SD or medians (interquartile ranges) for skewed variables or numbers (proportions) for categorical variables. *P* values were calculated from Student’s t tests for continuous variables and chi-square test for categorical variables

*BMI* body mass index, *HOMA-IR* homeostasis model assessment of insulin resistance, *HDL-C* high density lipoprotein cholesterol, *LDL-C* low density lipoprotein cholesterol

### Association of baseline FM/FFM ratio with risk of incident NAFLD by sex and obesity status

**Fig. 1:**
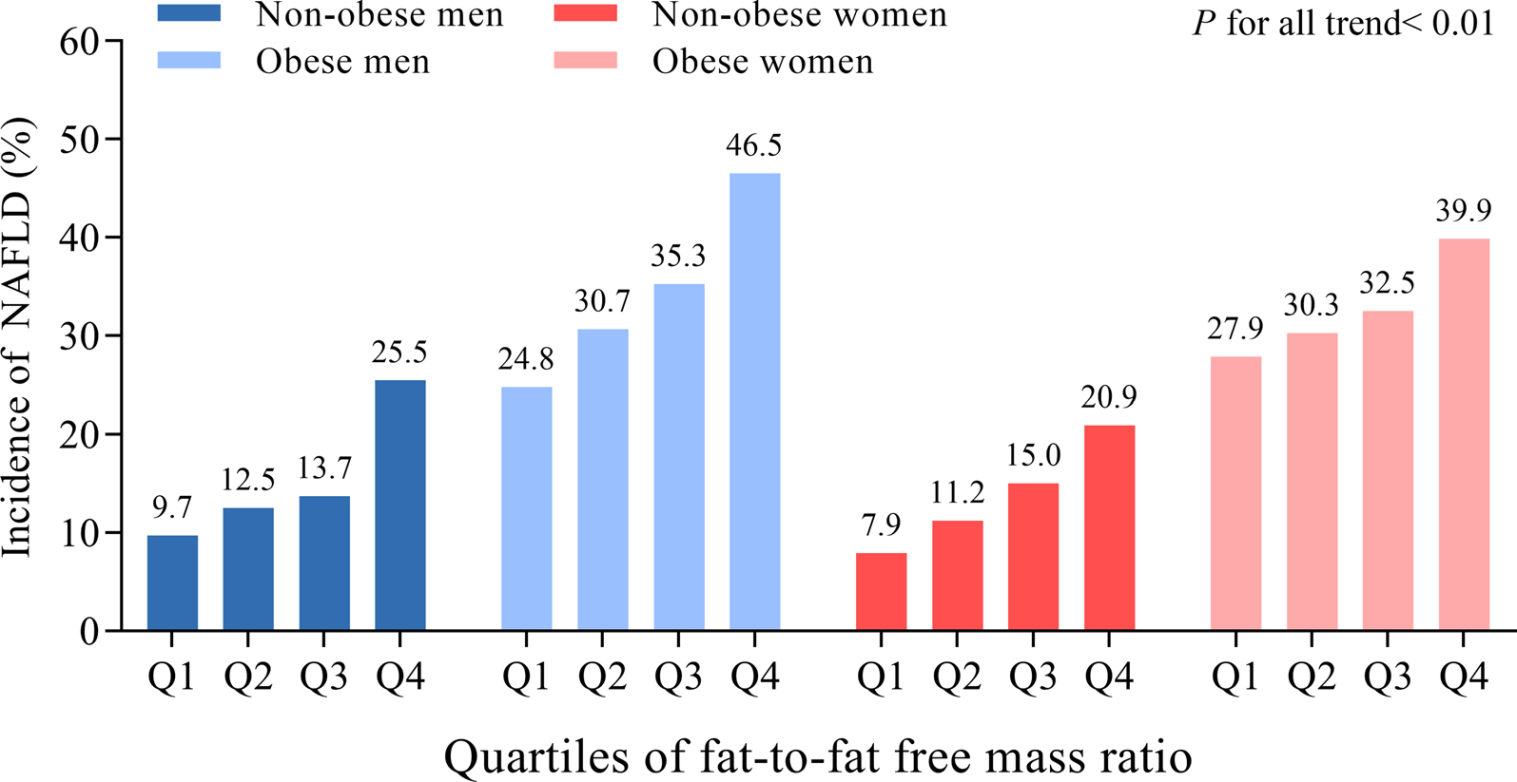
Incidence of NAFLD according to quartiles of FM/FFM by sex and obesity status

The incidence of NAFLD increased over quartiles of FM/FFM ratio in both non-obesity and obesity groups in men and women (*p* < 0.001) (Fig. 1).

The logistic regression model included age, smoking and drinking status, active physical activity, and education level as confounders showed that the FM/FFM ratio significantly associated with the risk of incident NAFLD in both sexes no matter obesity or not. In men, per one standard deviation (SD) increment of FM/FFM ratio was associated with 55% (95% CI 1.23–1.95) higher risk of incident NAFLD in non-obesity and 33% (95% CI 1.08–1.65) higher risk in obesity. In women, the corresponding value was 44% (95% CI 1.24–1.67) in non-obesity and 29% (95% CI 1.12–1.50) in obesity. Further adjusted for fasting glucose, HOMA-IR, triglycerides, LDL-C, HDL-C, and white blood cell counts, there was a reduction in the association of NAFLD risk with per one SD of FM/FFM ratio; however, it was still significant in non-obese women (OR 1.28, 95% CI 1.10–1.50, *p* = 0.002) and obese women (OR 1.22, 95% CI 1.04–1.42, *p* = 0.013), and marginally significant in non-obese and obese men (both *p* = 0.064) (Table 2). In the analysis without stratified participants according to obesity status (Additional File 1: Table S1) or sex (Additional File 1: Table S2), FM/FFM still associated with higher NAFLD risk in all sex and obesity subgroups after adjustment of previously described factors and sex or obesity status (see Additional File 1).

**Table 2:** Risks of developing NAFLD in relation to FM/FFM ratio by sex and obesity status

	Quartile 1	Quartile 2	Quartile 3	Quartile 4	*p* for trend	Per 1 SD increment	*p* value
Men (n = 1017)							
Non-obesity (n = 612)							
Model 1	Reference	1.32 (0.65–2.71)	1.47 (0.73–2.98)	3.17 (1.66–6.04)	< 0.001	1.59 (1.28–1.98)	< 0.001
Model 2		1.19 (0.57–2.48)	1.24 (0.60–2.57)	2.80 (1.44–5.42)	0.001	1.55 (1.23–1.95)	< 0.001
Model 3		0.89 (0.42–1.91)	0.92 (0.43–1.97)	1.61 (0.79–3.28)	0.127	1.27 (0.99–1.63)	0.064
Obesity (n = 405)							
Model 1	Reference	1.35 (0.73–2.50)	1.66 (0.90–3.04)	2.65 (1.46–4.81)	0.001	1.37 (1.12–1.69)	0.003
Model 2		1.40 (0.74–2.64)	1.51 (0.81–2.83)	2.52 (1.35–4.71)	0.004	1.33 (1.08–1.65)	0.009
Model 3		1.23 (0.64–2.39)	1.23 (0.64–2.38)	2.15 (1.11–4.18)	0.028	1.25 (0.99–1.58)	0.064
Women (n = 2402)							
Non-obesity (n = 1569)							
Model 1	Reference	1.47 (0.91–2.39)	2.06 (1.30–3.26)	3.08 (1.98–4.78)	< 0.001	1.44 (1.24–1.66)	< 0.001
Model 2		1.57 (0.96–2.57)	2.21 (1.38–3.54)	3.27 (2.07–5.16)	< 0.001	1.44 (1.24–1.67)	< 0.001
Model 3		1.29 (0.78–2.16)	1.77 (1.08–2.91)	2.35 (1.44–3.81)	< 0.001	1.28 (1.10–1.50)	0.002
Obesity (n = 833)							
Model 1	Reference	1.12 (0.74–1.72)	1.25 (0.82–1.90)	1.72 (1.14–2.59)	0.009	1.24 (1.08–1.44)	0.003
Model 2		1.20 (0.78–1.85)	1.29 (0.84–1.99)	1.93 (1.27–2.94)	0.002	1.29 (1.12–1.50)	0.001
Model 3		1.14 (0.73–1.79)	1.13 (0.72–1.79)	1.63 (1.05–2.55)	0.039	1.22 (1.04–1.42)	0.013

Data are odds ratio (OR) and 95% confidence interval (CI)

*P* values were calculated from the logistic regression models. Model 1 was crude model. Model 2 adjusted for age, current smoking, current drinking, active physical activity, and education level. Model 3 further adjusted for fasting glucose, HOMA-IR, triglycerides, LDL-C, HDL-C, white blood cells based on model 2

*FM/FFM* fat-to-fat free mass ratio, *OR* odds ratio, *CI* confidence interval, *SD* standard deviation, *HOMA-IR* homeostasis model assessment of insulin resistance, *HDL-C* high density lipoprotein cholesterol, *LDL-C* low density lipoprotein cholesterol

### Association of baseline FM/FFM ratio with risk of developing NAFLD with fibrosis by sex and obesity status

**Fig. 2:**
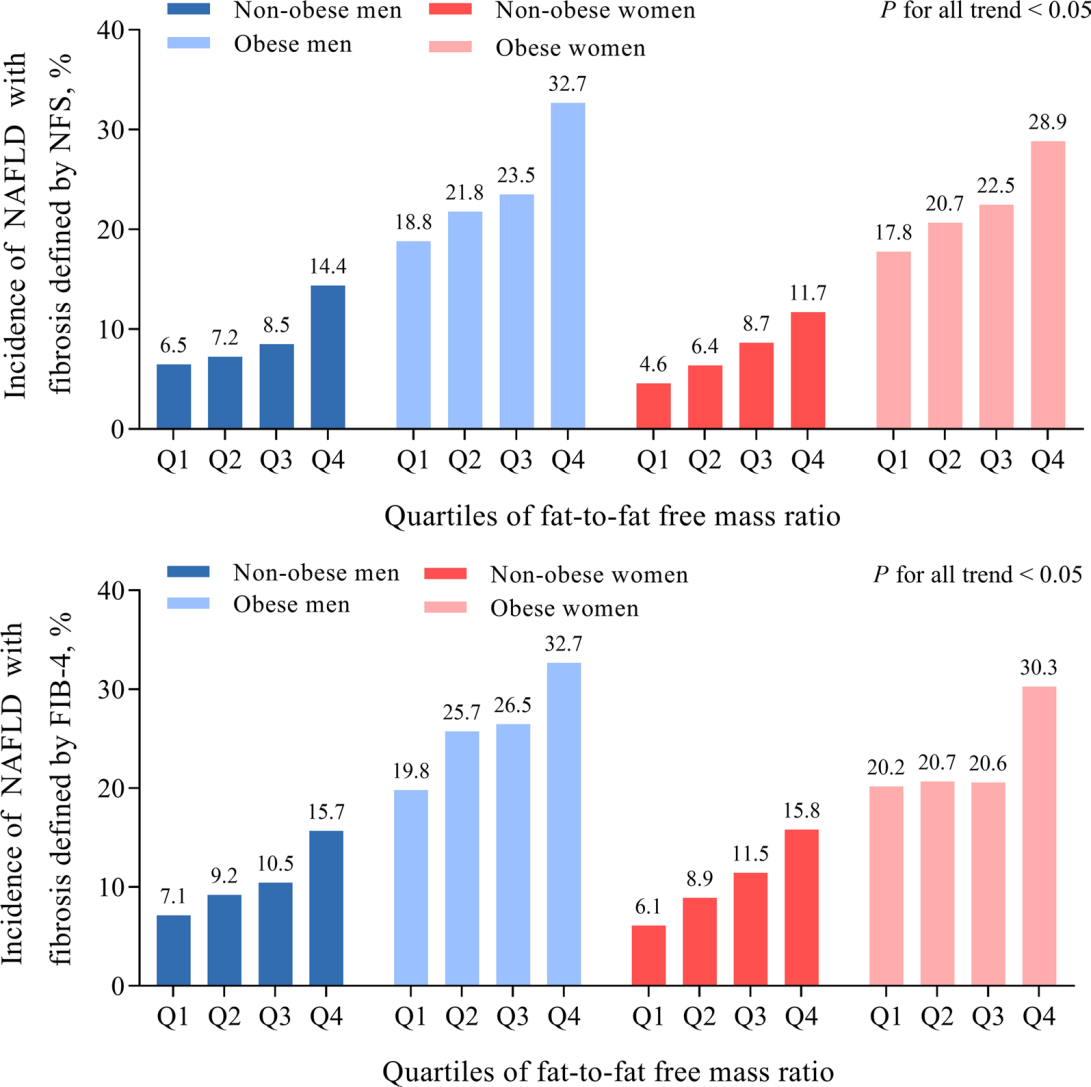
Incidence of NAFLD with fibrosis according to quartiles of FM/FFM by sex and obesity status

The incidence of NAFLD with fibrosis increased over quartiles of FM/FFM ratio in non-obesity and obesity in both sexes (*p* < 0.05) (Fig. 2). As shown in Table 3, in multivariate logistic regression models, the FM/FFM ratio was significantly associated with a higher risk of NAFLD with fibrosis, the adjusted ORs per SD increment of FM/FFM ratio were 1.42 (95% CI 1.07–1.89) for developing NAFLD with fibrosis assessed by NFS, and 1.47 (95% CI 1.13–1.92) for that by FIB-4 in non-obese men, and the corresponding risks within each subgroup were 1.41(95% CI 1.12–1.78) and 1.32 (95% CI 1.06–1.66) in obese men; 1.37(95% CI 1.15–1.63) and 1.39 (95% CI 1.19–1.63) in non-obese women; 1.29 (95% CI 1.10–1.51) and 1.27 (95% CI 1.08–1.49) in obese women. When further taking the effect of multiple metabolic and inflammatory indicators into account, FM/FFM ratio still associated with the risk of NAFLD with fibrosis in women, not in men. In the analysis without stratified participants according to obesity status (Additional File 1: Table S3) or sex (Additional File 1: Table S4), FM/FFM still associated with a higher NAFLD with fibrosis risk in all subgroups after adjustment of previously described factors and sex or obesity status (see Additional File 1).

**Table 3:** Associations of risk of developing NAFLD with fibrosis with FM/FFM by sex and obesity status

	Quartile 1	Quartile 2	Quartile 3	Quartile 4	*p* for trend	Per 1 SD increment	*p* value
Men (n = 1017)							
Non-obesity (n = 612)							
NAFLD with NFS ≥ − 1.455							
Model 1	Reference	1.12 (0.46–2.73)	1.34 (0.57–3.15)	2.42 (1.10–5.30)	0.018	1.36 (1.04–1.78)	0.024
Model 2		1.13 (0.45–2.82)	1.22 (0.50–3.01)	2.68 (1.19–6.03)	0.013	1.42 (1.07–1.89)	0.016
Model 3		0.76 (0.29–2.00)	0.91 (0.36–2.33)	1.60 (0.67–3.84)	0.170	1.20 (0.88–1.64)	0.257
NAFLD with FIB-4 ≥ 1.3							
Model 1	Reference	1.32 (0.58–3.01)	1.52 (0.68–3.39)	2.42 (1.14–5.13)	0.017	1.39 (1.08–1.79)	0.010
Model 2		1.49 (0.64–3.43)	1.45 (0.63–3.35)	2.78 (1.28–6.06)	0.011	1.47 (1.13–1.92)	0.004
Model 3		1.09 (0.46–2.60)	1.10 (0.46–2.61)	1.72 (0.75–3.96)	0.181	1.25 (0.94–1.67)	0.130
Obesity (n = 405)							
NAFLD with NFS ≥ − 1.455							
Model 1	Reference	1.20 (0.61–2.39)	1.33 (0.68–2.61)	2.09 (1.09–4.01)	0.024	1.35 (1.08–1.69)	0.009
Model 2		1.42 (0.70–2.87)	1.48 (0.73–2.99)	2.59 (1.30–5.16)	0.008	1.41 (1.12–1.78)	0.003
Model 3		1.42 (0.68–2.98)	1.41 (0.66–2.98)	2.69 (1.28–5.65)	0.011	1.40 (1.08–1.80)	0.010
NAFLD with FIB-4 ≥ 1.3							
Model 1	Reference	1.40 (0.72–2.72)	1.46 (0.76–2.81)	1.97 (1.03–3.74)	0.045	1.25 (1.00–1.55)	0.046
Model 2		1.71 (0.86–3.39)	1.62 (0.81–3.22)	2.53 (1.28–5.03)	0.013	1.32 (1.06–1.66)	0.016
Model 3		1.66 (0.81–3.41)	1.47 (0.71–3.04)	2.46 (1.19–5.09)	0.029	1.28 (1.00–1.64)	0.053
Women (n = 2402)							
Non-obesity (n = 1569)							
NAFLD with NFS ≥ − 1.455							
Model 1	Reference	1.42 (0.76–2.64)	1.97 (1.09–3.55)	2.76 (1.57–4.86)	< 0.001	1.37 (1.15–1.63)	< 0.001
Model 2		1.59 (0.83–3.04)	2.28 (1.22–4.23)	3.07 (1.68–5.62)	< 0.001	1.37 (1.15–1.63)	< 0.001
Model 3		1.38 (0.72–2.68)	1.91 (1.01–3.61)	2.40 (1.27–4.51)	0.003	1.26 (1.06–1.51)	0.009
NAFLD with FIB-4 ≥ 1.3							
Model 1	Reference	1.50 (0.88–2.58)	1.98 (1.18–3.32)	2.88 (1.76–4.72)	< 0.001	1.39 (1.19–1.63)	< 0.001
Model 2		1.63 (0.93–2.84)	2.21 (1.29–3.78)	3.14 (1.87–5.28)	< 0.001	1.39 (1.19–1.63)	< 0.001
Model 3		1.38 (0.79–2.44)	1.77 (1.02–3.07)	2.27 (1.32–3.92)	0.002	1.25 (1.06–1.47)	0.008
Obesity (n = 833)							
NAFLD with NFS ≥ − 1.455							
Model 1	Reference	1.20 (0.74–1.96)	1.34 (0.83–2.17)	1.87 (1.18–2.98)	0.007	1.22 (1.05–1.43)	0.012
Model 2		1.38 (0.83–2.29)	1.52 (0.92–2.52)	2.25 (1.39–3.66)	0.001	1.29 (1.10–1.51)	0.002
Model 3		1.36 (0.81–2.28)	1.40 (0.83–2.35)	2.03 (1.23–3.35)	0.007	1.25 (1.06–1.47)	0.001
NAFLD with FIB-4 ≥ 1.3							
Model 1	Reference	1.03 (0.64–1.66)	1.02 (0.64–1.65)	1.72 (1.10–2.69)	0.021	1.20 (1.03–1.40)	0.023
Model 2		1.18 (0.72–1.93)	1.15 (0.70–1.90)	2.06 (1.29–3.29)	0.003	1.27 (1.08–1.49)	0.004
Model 3		1.13 (0.68–1.88)	1.03 (0.61–1.73)	1.89 (1.16–3.07)	0.017	1.23 (1.04–1.45)	0.015

Data are odds ratio (OR) and 95% confidence interval (CI)

*P* values were calculated from the logistic regression models. Model 1 was crude model. Model 2 adjusted for age, current smoking, current drinking, active physical activity, and education level. Model 3 further adjusted for fasting glucose, HOMA-IR, triglycerides, LDL-C, HDL-C, white blood cells based on model 2

*FM/FFM* fat-to-fat free mass ratio, *OR* odds ratio, *CI* confidence interval, *SD* standard deviation, *BMI* body mass index, *HOMA-IR* homeostasis model assessment of insulin resistance, *HDL-C* high density lipoprotein cholesterol, *LDL-C* low density lipoprotein cholesterol

### Mediation analysis of metabolic and inflammatory factors in the association between the FM/FFM ratio and incident NAFLD in obese and non-obese individuals

**Fig. 3:**
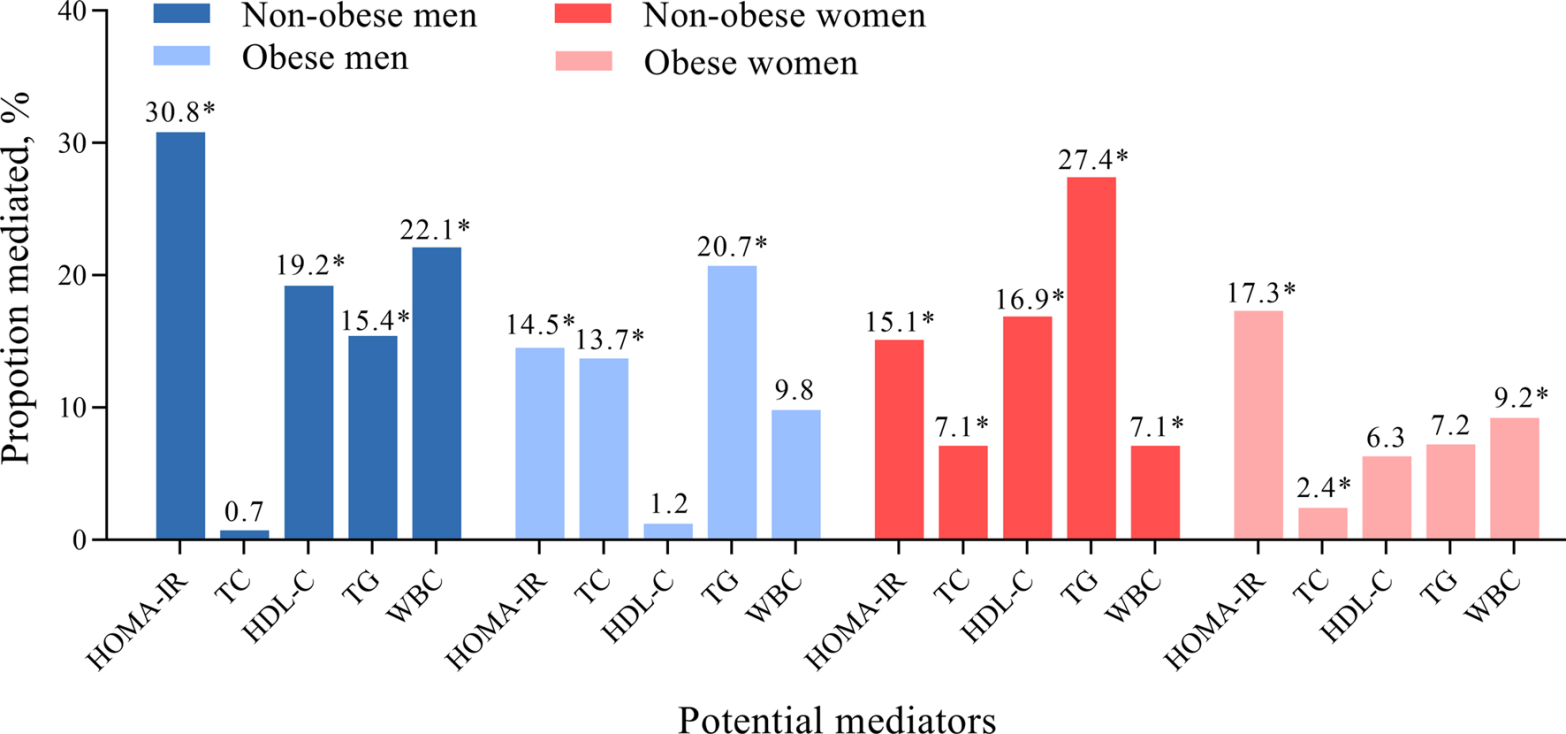
Mediation analysis of the associations of NAFLD risk with FM/FFM by sex and obesity status. *The mediation effect of potential mediator reached a statistically significant level. Triglycerides and HOMA‐IR were log‐transformed before analysis. The covariates were age, current smoking, current drinking, active physical activity, and education level. The mediating effects of each mediator were tested by applying the counterfactual mediation method. Controlled direct effect (CDE) represented the effect of exposure on the outcome via pathways that do not involve the mediator, natural indirect effects (NIE) represented the effect of exposure on the outcome operating through the mediators, and the total effect represented the sum of all ways of the effect of exposure on the outcome. The proportions of the association between the exposure and the outcome which is mediated by the mediators were calculated using the following formula: (CDE * (NIE − 1))/(CDE * NIE − 1). *FM/FFM* fat-to fat free mass ratio, *HOMA-IR* homeostasis model assessment of insulin resistance, *HDL-C* high density lipoprotein cholesterol, *WBC* white blood cells, *TC* total cholesterol, *TG* triglycerides

As shown in Fig. 3, in men, HOMA-IR and triglycerides mediated the associations between FM/FFM ratio and NAFLD risk significantly in both non-obesity and obesity, with a proportion of 30.8%, 15.4% in non-obesity and 14.5%, 20.7% in obesity. HDL-C (19.2%) and white blood cells (22.1%) mediated the associations significantly only in non-obesity and total cholesterol (13.7%) only in obesity. In women, the mediated factors which reached a significant level in both groups were HOMA-IR, HDL-C, and white blood cells, the relative proportions mediated were 15.1%, 16.9%, 7.1% in non-obesity and 17.3%, 6.3%, 9.2% in obesity. Triglycerides (27.4%) and total cholesterol (7.1%) mediated the associations significantly only in non-obese women. We also considered other potential mediators included fasting glucose, LDL-C, and systolic blood pressure, but the mediated effect of these factors did not reach a significant level in any sex and obesity subgroups (Table 4).

**Table 4:** Adjusted direct and indirect associations of FM/FFM with NAFLD by sex and obesity status

Potential mediators	Non-obesity				Obesity			
	Total effect	Controlled direct effect	Natural indirect effect	Proportion mediated (%)	Total effect	Controlled direct effect	Natural indirect effect	Proportion mediated (%)
Men								
HOMA-IR	1.58 (1.20–2.06)	1.41 (1.08–1.82)	1.13 (1.05–1.24)	30.8	1.36 (1.08–1.68)	1.31 (1.03–1.62)	1.04 (1.00–1.09)	14.5
Fasting glucose	1.57 (1.21–2.03)	1.57 (1.21–2.02)	1.00 (0.99–1.02)	0.9	1.36 (1.09–1.72)	1.36 (1.09–1.73)	1.00 (0.98–1.02)	− 1.0
Total cholesterol	1.58 (1.25–1.96)	1.57 (1.23–1.94)	1.00 (0.96–1.05)	0.7	1.36 (1.09–1.69)	1.31 (1.04–1.63)	1.04 (1.00–1.09)	13.7
Triglycerides	1.59 (1.24–2.11)	1.50 (1.18–1.96)	1.06 (1.01–1.15)	15.4	1.35 (1.07–1.7)	1.28 (1.01–1.63)	1.06 (1.01–1.13)	20.7
HDL-C	1.60 (1.24–2.03)	1.48 (1.16–1.92)	1.08 (1.02–1.17)	19.2	1.36 (1.08–1.72)	1.36 (1.08–1.71)	1.00 (0.99–1.02)	1.2
LDL-C	1.56 (1.23–1.98)	1.53 (1.20–1.93)	1.02 (0.97–1.08)	5.5	1.36 (1.08–1.76)	1.34 (1.06–1.74)	1.02 (0.99–1.05)	5.6
SBP	1.59 (1.24–2.04)	1.53 (1.20–1.93)	1.04 (0.99–1.09)	9.5	1.36 (1.08–1.75)	1.34 (1.06–1.70)	1.02 (0.99–1.05)	6.0
White blood cells	1.55 (1.17–1.98)	1.43 (1.09–1.79)	1.09 (1.02–1.16)	22.1	1.40 (1.12–1.77)	1.37 (1.08–1.74)	1.03 (0.99–1.09)	9.8
Women								
HOMA-IR	1.48 (1.23–1.79)	1.40 (1.17–1.70)	1.05 (1.02–1.09)	15.1	1.30 (1.11–1.52)	1.25 (1.06–1.45)	1.04 (1.01–1.08)	17.3
Fasting glucose	1.45 (1.21–1.73)	1.46 (1.22–1.73)	1.00 (0.99–1.00)	− 0.7	1.30 (1.11–1.54)	1.31 (1.12–1.57)	0.99 (0.97–1.00)	− 4.1
Total cholesterol	1.46 (1.21–1.78)	1.43 (1.20–1.73)	1.02 (1.00–1.05)	7.1	1.29 (1.10–1.51)	1.29 (1.10–1.50)	1.01 (1.00–1.02)	2.4
Triglycerides	1.47 (1.23–1.77)	1.34 (1.12–1.63)	1.10 (1.06–1.14)	27.4	1.30 (1.11–1.53)	1.28 (1.09–1.49)	1.02 (0.99–1.05)	7.2
HDL-C	1.49 (1.25–1.81)	1.41 (1.18–1.70)	1.06 (1.03–1.10)	16.9	1.31 (1.11–1.56)	1.29 (1.10–1.52)	1.02 (0.99–1.04)	6.3
LDL-C	1.46 (1.23–1.77)	1.43 (1.20–1.74)	1.02 (0.99–1.05)	7.0	1.30 (1.12–1.52)	1.29 (1.11–1.51)	1.01 (1.00–1.02)	2.8
SBP	1.46 (1.21–1.75)	1.46 (1.21–1.76)	1.00 (0.98–1.02)	0.9	1.30 (1.12–1.56)	1.29 (1.10–1.53)	1.01 (1.00–1.03)	4.8
White blood cells	1.48 (1.23–1.80)	1.44 (1.20–1.75)	1.02 (1.00–1.05)	7.1	1.29 (1.09–1.51)	1.26 (1.07–1.47)	1.02 (1.00–1.05)	9.2

Data are odds ratio (OR) and 95% confidence interval (CI)

The covariates were age, current smoking, current drinking, active physical activity, and education level

The mediating effects of each mediator were tested by applying the counterfactual mediation method. Controlled direct effect (CDE) represented the effect of exposure on the outcome via pathways that do not involve the mediator, natural indirect effects (NIE) represented the effect of exposure on the outcome operating through the mediators, and the total effect represented the sum of all ways of the effect of exposure on the outcome. The proportions of the association between the exposure and the outcome which is mediated by the mediators were calculated using the following formula: (CDE * (NIE − 1))/(CDE * NIE − 1)

*FM/FFM* fat-to-fat free mass ratio, *OR* odds ratio, *CI* confidence interval, *SD* standard deviation, *HOMA-IR* homeostasis model assessment of insulin resistance, *HDL-C* high density lipoprotein cholesterol, *LDL-C* low density lipoprotein cholesterol, *SBP* systolic blood pressure

## Discussion

In this prospective cohort study of 3419 community-dwelling Chinese adults, we found that the FM/FFM ratio significantly associated with a higher risk of developing NAFLD and a higher probability of fibrosis in both non-obese and obese individuals. Mediation analysis showed that there may be a small difference when it comes to the mediators between FM/FFM ratio and risk of NAFLD according to sex and obesity status.

The presence of excess fat mass is a well-established risk factor for NAFLD in both non-obese and obese individuals. A cross-sectional study in the Netherlands population reported that an increase of total fat mass was significantly associated with a higher risk of NAFLD both in participants with normal weight and in overweight [27]. A longitudinal study found that higher fat percentage at baseline may be a predictor of incident NAFLD across all obesity spectrum [28]. At the same time, emerging evidence suggested that the substantial loss of muscle mass, the main component of fat-free mass, termed as ‘sarcopenia’, had a negative effect on the risk of NAFLD and fibrosis independent of obesity. Lee YH et al. demonstrated that individuals with sarcopenia had a higher risk of NAFLD and fibrosis independent from the status of obesity compared with individuals with a preserved muscle mass using a nationally representative study [29]. A cross-sectional study that included participants aged 18–80 years found that sarcopenia was significantly correlated with a higher risk of NAFLD in non-obese individuals [30]. Besides, a longitudinal study reported that a progressive increase in fat mass and a loss of skeletal muscle mass with aging were significantly associated with incident NAFLD, and this association was more prominent in non-obese individuals [31].

The construction of the FM/FFM ratio integrated the metabolic effect of both fat mass and fat-free mass. This indicator was regarded as a measurement of sarcopenic obesity, a body composition-defined definition using to describe individuals with both evidence of muscle loss and excess adiposity [17, 32]. It has been reported that individuals had the highest risk when there was a concurrence of sarcopenia and obesity [30]. Prevalence of sarcopenic obesity increased over quintile of serum gamma-glutamyl transferase in community-dwelling older adults [33]. Besides, studies have linked sarcopenia obesity with insulin resistance, dyslipidemia, and T2D, which all implicated in the development of NAFLD [34]. Meanwhile, it has been reported that the FM/FFM ratio was positively associated with insulin resistance in the general population and liver fat accumulation, which was an important pathological feature of NAFLD, in patients with T2D directly [18, 35]. Besides, studies also suggested that the FM/FFM ratio was capable to be regarded as a novel predictor of metabolic syndrome and glucose metabolic disorder, which both were associated closely with NAFLD [18–20, 36]. Combined these studies with the findings of our study, the FM/FFM ratio could be a useful predictor of the risk of developing NAFLD among different obesity status.

We observed a small difference in sex and obesity status when it comes to the mediators between FM/FFM ratio and NAFLD risk. Insulin resistance was a major mediator in all subgroups, especially in non-obese men. HDL-C only mediated the association in non-obese participants. White blood cells, a marker of inflammation level, was also a significant mediator in women and non-obese men.

Sex difference exists in all the possible pathogenesis pathways of NAFLD including systemic inflammation, insulin resistance, oxidative stress, and triglycerides synthesis [37]. A previous study reported that the association between metabolic risk factors and NAFLD was 1.5 to 2‐folds stronger in men than in women [38]. Sex is also a major determinant of body composition. Women predispose to store fat in the subcutaneous adipose tissue, whereas men tend to store fat in the visceral adipose tissue [39]. The different associations between subcutaneous and visceral adipose tissue and cardiometabolic disease risk are also possible reasons for the difference in mediating factors between body composition and NAFLD [40].

Obesity status modified the relationship between metabolic risk factors and NAFLD [38]. The differences in metabolic risk factors of NAFLD between non-obese and obese people are not well elucidated. Compared with obese NAFLD patients, non-obese NAFLD patients have a lower proportion of metabolic disorders [41] but a severe histological picture similar to obese patients [42]. One potential explanation might be due to a substantial difference in fecal and blood microbiota profiles between obese and lean individuals with NAFLD [43]. Individuals who carry the PNPLA3 rs738409 gene polymorphism have a greater risk of developing NAFLD [44]; in addition, a meta-analysis showed that the PNPLA3 rs738409 gene polymorphism is more prevalent in non‐obese NAFLD patients than that in obese NAFLD patients [41]. Besides, compared with obese NAFLD patients, non-obese individuals with NAFLD had a substantially lower polyunsaturated fatty acid intake, which can ameliorate the activity of NAFLD through reducing hepatic TNF-α expression and improving insulin resistance in animal models [45]. Further investigations focused on the relationship between metabolic factors and NAFLD among different obesity status may be helpful to understand diverse NAFLD pathogenesis.

There were several strengths of our study including a prospective cohort design, a well-defined community setting, and a highly homogeneous population. The limitations should also be acknowledged. Firstly, body compositions were measured by BIA, instead of dual-energy X-ray absorptiometry, which was considered a more reliable method for body composition assessment [46]. However, BIA is a convenient body composition assessment tool possessing the merits of low cost, ease of use, and non-invasive nature, so it is a more practical method in health practice. Besides, the Asian Working Group of Sarcopenia supported the use of BIA in community-based settings [47]. Secondly, due to the unavailable data of hepatic biopsy, we used ultrasound imaging and several predicting models of fibrosis that have been well validated to define the probability of fibrosis in NAFLD patients [25]. Thirdly, we could not assess the contribution of the fat distribution in the association of FM/FFM ratio with NAFLD, due to lack of body fat distribution measurement data. A growing body of literature suggested that increased visceral fat mass is a strong determinant of cardiometabolic diseases, while subcutaneous abdominal fat mass shows a weak or non-significant association [40]. It is helpful to take body fat distribution into account when stratifying the risk of cardiometabolic diseases for a given fat mass in future studies. Finally, our population was limited to middle-aged and elderly Chinese, a wider age range of study populations will provide more important information about the usefulness of the FM/FFM ratio in other age and ethnicity groups.

## Conclusions

In conclusion, we found that the FM/FFM ratio was significantly associated with the risk of developing NAFLD and fibrosis in both non-obese and obese individuals in this community-based prospective study. Our study hints that there may be differences in factors mediated the association between FM/FFM ratio and NAFLD risk according to different obesity status.

## Supplementary Information

**Addition file 1: Table S1**. Associations of risk of NAFLD with fat-to-fat free mass ratio by sex. **Table S2**. Associations of risk of NAFLD with fat-to-fat free mass ratio by obesity status. **Table S3**. Associations of risk of NAFLD with fibrosis of fat‐to‐fat free mass ratio by sex. **Table S4**. Associations of risk of NAFLD with fibrosis of fat‐to‐fat free mass ratio by obesity status.

## Data Availability

Restrictions apply to some or all the availability of data generated or analyzed during this study to preserve patient confidentiality or because they were used under license. The corresponding author will on request detail the restrictions and any conditions under which access to some data may be provided.

## Abbreviations

NAFLD: Non-alcoholic fatty liver disease
FM/FFM: Fat mass to fat-free mass ratio
OR: Odds ratio
CI: Confidence interval
NASH: Nonalcoholic steatohepatitis
T2D: Type 2 diabetes
BMI: Body mass index
HOMA-IR: Homeostasis model assessment of insulin resistance
LDL-C: Low density lipoprotein cholesterol
HDL-C: High density lipoprotein cholesterol
ALT: Alanine aminotransferase
AST: Aspartate aminotransferase
GGT: γ‐Glutamyl transferase
BIA: Bioelectrical impedance analysis
NFS: Non-alcoholic fatty liver disease fibrosis score
FIB-4: Fibrosis-4 score
WBC: White blood cells
SBP: Systolic blood pressure
TG: Triglycerides
TC: Total cholesterol
