# Supplementary material for: Fat mass to fat-free mass ratio and the risk of non-alcoholic fatty liver disease and fibrosis in non-obese and obese individuals
Source: Nutr Metab (Lond). 2021 Feb 19;18:21. doi: 10.1186/s12986-021-00551-6 (PMC7893940; doi:10.1186/s12986-021-00551-6)
Supplement: Supplementary file 1 — Addition file 1: Table S1. Associations of risk of NAFLD with fat-to-fat free mass ratio by sex. Table S2. Associations of risk of NAFLD with fat-to-fat free mass ratio by obesity status. Table S3. Associations of risk of NAFLD with fibrosis of fat‐to‐fat free mass ratio by sex. Table S4. Associations of risk of NAFLD with fibrosis of fat‐to‐fat free mass ratio by obesity status. [file 12986_2021_551_MOESM1_ESM.docx]

**Supplementary table 1**. Associations of risk of NAFLD with fat-to-fat free mass ratio by sex.

|  | Quartile 1 | Quartile 2 | Quartile 3 | Quartile 4 | *p* for trend | Per 1 SD increment | *p* value |
| --- | --- | --- | --- | --- | --- | --- | --- |
| Men (n = 1,017) |  |  |  |  |  |  |  |
| Model 1 | Reference | 1.23 (0.73 - 2.07) | 2.77 (1.73 - 4.44) | 4.69 (2.97 - 7.41) | <0.001 | 1.81 (1.55 - 2.10) | <0.001 |
| Model 2 |  | 1.02 (0.60 - 1.73) | 2.01 (1.21 - 3.32) | 2.75 (1.61 - 4.72) | <0.001 | 1.49 (1.24 - 1.80) | <0.001 |
| Model 3 |  | 0.79 (0.45 - 1.36) | 1.31 (0.77 - 2.23) | 1.69 (0.95 – 3.00) | 0.015 | 1.31 (1.07 - 1.59) | 0.008 |
|  |  |  |  |  |  |  |  |
| Women (n = 2,402) |  |  |  |  |  |  |  |
| Model 1 | Reference | 2.14 (1.49 - 3.08) | 3.25 (2.30 - 4.59) | 5.62 (4.02 - 7.86) | <0.001 | 1.72 (1.55 - 1.90) | <0.001 |
| Model 2 |  | 2.03 (1.40 - 2.93) | 2.61 (1.80 - 3.80) | 3.63 (2.40 - 5.48) | <0.001 | 1.44 (1.27 - 1.63) | <0.001 |
| Model 3 |  | 1.68 (1.15 - 2.47) | 2.03 (1.38 - 2.99) | 2.49 (1.62 - 3.84) | <0.001 | 1.30 (1.14 - 1.48) | <0.001 |

Data are odds ratio (OR) and 95% confidence interval (CI).

*P* values were calculated from the logistic regression models. Model 1 was crude model. Model 2 adjusted for age, obesity status, current smoking, current drinking, active physical activity, and education level. Model 3 further adjusted for fasting glucose, HOMA-IR, triglycerides, LDL-C, HDL-C, white blood cells based on model 2.

The range of quartiles of fat-to-fat free mass ratio were < 0.25, 0.25 - 0.31, 0.31 - 0.38, > 0.38 in men and < 0.40, 0.40 - 0.49, 0.49 - 0.59, > 0.59 in women.

Abbreviation: OR, odds ratio; CI, confidence interval; SD, standard deviation; HOMA-IR, homeostasis model assessment of insulin resistance; HDL-C, high density lipoprotein cholesterol; LDL-C, low density lipoprotein cholesterol.

**Supplementary table 2.** Associations of risk of NAFLD with fat-to-fat free mass ratio by obesity status.

|  | Quartile 1 | Quartile 2 | Quartile 3 | Quartile 4 | *p* for trend |
| --- | --- | --- | --- | --- | --- |
| Non-obesity (n = 2,181) |  |  |  |  |  |
| Model 1 | Reference | 1.42 (0.95 - 2.12) | 1.87 (1.27 - 2.74) | 3.10 (2.16 - 4.46) | <0.001 |
| Model 2 |  | 1.47 (0.98 - 2.21) | 1.90 (1.28 - 2.81) | 3.21 (2.21 - 4.66) | <0.001 |
| Model 3 |  | 1.17 (0.77 - 1.78) | 1.45 (0.97 - 2.18) | 2.11 (1.42 - 3.15) | <0.001 |
|  |  |  |  |  |  |
| Obesity (n = 1,238) |  |  |  |  |  |
| Model 1 | Reference | 1.19 (0.84 - 1.69) | 1.37 (0.97 - 1.93) | 1.98 (1.41 - 2.77) | <0.001 |
| Model 2 |  | 1.25 (0.88 - 1.79) | 1.37 (0.96 - 1.95) | 2.08 (1.47 - 2.94) | <0.001 |
| Model 3 |  | 1.12 (0.78 - 1.62) | 1.14 (0.79 - 1.64) | 1.70 (1.18 - 2.45) | 0.005 |

Data are odds ratio (OR) and 95% confidence interval (CI). Quartiles range of FM/FFM were defined by sex.

*P* values were calculated from the logistic regression models. Model 1 was crude model. Model 2 adjusted for age, sex, current smoking, current drinking, active physical activity, and education level. Model 3 further adjusted for fasting glucose, HOMA-IR, triglycerides, LDL-C, HDL-C, white blood cells based on model 2.

Abbreviation: OR, odds ratio; CI, confidence interval; SD, standard deviation; BMI, body mass index; HOMA-IR, homeostasis model assessment of insulin resistance; HDL-C, high density lipoprotein cholesterol; LDL-C, low density lipoprotein cholesterol.

**Supplementary table 3.** Associations of risk of NAFLD with fibrosis of fat‐to‐fat free mass ratio by sex.

|  | Quartile 1 | Quartile 2 | Quartile 3 | Quartile 4 | *p* for trend | Per 1 SD increment | *p* value |
| --- | --- | --- | --- | --- | --- | --- | --- |
| Men (n = 1,017) | | | | | | |  |
| NAFLD with NFS ≥ −1.455 | | | | | | |  |
| Model 1 | Reference | 1.50 (0.80 - 2.80) | 2.89 (1.62 - 5.13) | 4.42 (2.53 - 7.71) | <0.001 | 1.72 (1.45 - 2.03) | <0.001 |
| Model 2 |  | 1.25 (0.65 - 2.40) | 2.20 (1.18 - 4.09) | 2.85 (1.47 - 5.50) | <0.001 | 1.49 (1.21 - 1.82) | <0.001 |
| Model 3 |  | 0.99 (0.50 - 1.93) | 1.57 (0.82 - 3.02) | 2.04 (1.01 - 4.10) | 0.014 | 1.39 (1.11 - 1.74) | 0.004 |
| NAFLD with FIB-4 ≥ 1.3 | | | | | | |  |
| Model 1 | Reference | 1.35 (0.76 - 2.39) | 2.51 (1.48 - 4.25) | 3.60 (2.16 - 6.00) | <0.001 | 1.64 (1.40 - 1.93) | <0.001 |
| Model 2 |  | 1.20 (0.66 - 2.17) | 2.01 (1.14 - 3.56) | 2.39 (1.30 - 4.40) | 0.002 | 1.44 (1.18 - 1.75) | <0.001 |
| Model 3 |  | 0.95 (0.52 - 1.74) | 1.43 (0.79 - 2.60) | 1.65 (0.87 - 3.16) | 0.057 | 1.32 (1.06 - 1.64) | 0.012 |
|  |  |  |  |  |  |  |  |
| Women (n = 2,402) | | | | | | |  |
| NAFLD with NFS ≥ −1.455 | | | | | | |  |
| Model 1 | Reference | 2.22 (1.41 - 3.51) | 3.07 (1.97 - 4.76) | 5.94 (3.91 - 9.02) | <0.001 | 1.69 (1.51 - 1.89) | <0.001 |
| Model 2 |  | 2.11 (1.31 - 3.4) | 2.35 (1.45 - 3.83) | 3.48 (2.07 - 5.86) | <0.001 | 1.38 (1.21 - 1.59) | <0.001 |
| Model 3 |  | 1.86 (1.15 - 3.01) | 1.96 (1.19 - 3.23) | 2.64 (1.54 - 4.52) | <0.001 | 1.29 (1.12 - 1.49) | <0.001 |
| NAFLD with FIB-4 ≥ 1.3 | | | | | | |  |
| Model 1 | Reference | 2.15 (1.43 - 3.21) | 2.73 (1.85 - 4.04) | 4.54 (3.12 - 6.61) | <0.001 | 1.56 (1.41 - 1.74) | <0.001 |
| Model 2 |  | 2.10 (1.39 - 3.19) | 2.39 (1.56 - 3.67) | 3.37 (2.11 - 5.37) | <0.001 | 1.38 (1.21 - 1.58) | <0.001 |
| Model 3 |  | 1.82 (1.19 - 2.77) | 1.92 (1.24 - 2.98) | 2.45 (1.51 - 3.97) | <0.001 | 1.28 (1.11 - 1.47) | <0.001 |

Data are odds ratio (OR) and 95% confidence interval (CI).

*P* values were calculated from the logistic regression models. Model 1 was crude model. Model 2 adjusted for age, obesity status, current smoking, current drinking, active physical activity, and education level. Model 3 further adjusted for fasting glucose, HOMA-IR, triglycerides, LDL-C, HDL-C, white blood cells based on model 2. The range of quartiles of fat-to-fat free mass ratio were < 0.25, 0.25 - 0.31, 0.31 - 0.38, > 0.38 in men and < 0.40, 0.40 - 0.49, 0.49 - 0.59, > 0.59 in women. Abbreviation: OR, odds ratio; CI, confidence interval; SD, standard deviation; HOMA-IR, homeostasis model assessment of insulin resistance; HDL-C, high density lipoprotein cholesterol; LDL-C, low density lipoprotein cholesterol.

**Supplementary table 4.** Associations of risk of NAFLD with fibrosis of fat‐to‐fat free mass ratio by obesity status.

|  | Quartile 1 | Quartile 2 | Quartile 3 | Quartile 4 | *p* for trend |
| --- | --- | --- | --- | --- | --- |
| Non-obesity (n = 2,181) |  |  |  |  |  |
| NAFLD with NFS ≥ −1.455 |  |  |  |  |  |
| Model 1 | Reference | 1.31 (0.79 - 2.18) | 1.74 (1.07 - 2.83) | 2.64 (1.67 - 4.17) | <0.001 |
| Model 2 |  | 1.42 (0.84 - 2.41) | 1.89 (1.15 - 3.13) | 2.99 (1.85 - 4.83) | <0.001 |
| Model 3 |  | 1.17 (0.69 – 2.00) | 1.52 (0.91 - 2.55) | 2.14 (1.29 - 3.54) | 0.001 |
| NAFLD with FIB-4 ≥ 1.3 |  |  |  |  |  |
| Model 1 | Reference | 1.45 (0.92 - 2.27) | 1.84 (1.19 - 2.83) | 2.74 (1.81 - 4.13) | <0.001 |
| Model 2 |  | 1.58 (1.00 - 2.51) | 1.97 (1.26 - 3.09) | 3.07 (2.00- 4.71) | <0.001 |
| Model 3 |  | 1.30 (0.81 - 2.08) | 1.54 (0.97 - 2.44) | 2.10 (1.34 - 3.31) | <0.001 |
|  |  |  |  |  |  |
| Obesity (n =1,238) |  |  |  |  |  |
| NAFLD with NFS ≥ −1.455 |  |  |  |  |  |
| Model 1 | Reference | 1.20 (0.81 - 1.79) | 1.34 (0.90 - 1.98) | 1.95 (1.33 - 2.84) | <0.001 |
| Model 2 |  | 1.39 (0.92 - 2.09) | 1.52 (1.01 - 2.29) | 2.34 (1.58 - 3.46) | <0.001 |
| Model 3 |  | 1.31 (0.86 - 1.99) | 1.33 (0.87 - 2.04) | 2.05 (1.36 - 3.08) | <0.001 |
| NAFLD with FIB-4 ≥ 1.3 |  |  |  |  |  |
| Model 1 | Reference | 1.15 (0.78 - 1.69) | 1.16 (0.79 - 1.70) | 1.80 (1.24 - 2.60) | 0.002 |
| Model 2 |  | 1.33 (0.89 - 1.99) | 1.31 (0.87 - 1.96) | 2.18 (1.48 - 3.19) | <0.001 |
| Model 3 |  | 1.24 (0.82 - 1.86) | 1.13 (0.75 - 1.72) | 1.91 (1.28 - 2.85) | 0.003 |

Data are odds ratio (OR) and 95% confidence interval (CI). Quartiles range of FM/FFM were defined by sex.

*P* values were calculated from the logistic regression models. Model 1 was crude model. Model 2 adjusted for age, sex, current smoking, current drinking, active physical activity, and education level. Model 3 further adjusted for fasting glucose, HOMA-IR, triglycerides, LDL-C, HDL-C, white blood cells based on model 2.

Abbreviation: OR, odds ratio; CI, confidence interval; SD, standard deviation; BMI, body mass index; HOMA-IR, homeostasis model assessment of insulin resistance; HDL-C, high density lipoprotein cholesterol; LDL-C, low density lipoprotein cholesterol.
